# Defective phagocyte association during infection of *Galleria mellonella* with *Yersinia pseudotuberculosis* is detrimental to both insect host and microbe

**DOI:** 10.1080/21505594.2021.1878672

**Published:** 2021-02-08

**Authors:** Anne Marie Krachler, Natalie Sirisaengtaksin, Pauline Monteith, C. E. Timothy Paine, Christopher J. Coates, Jenson Lim

**Affiliations:** a Department of Microbiology and Molecular Genetics, University of Texas McGovern Medical School at Houston, Houston, TX, USA; b Biological and Environmental Sciences, University of Stirling, Stirling, UK; c School of Environmental and Rural Sciences, University of New England, Armidale, Australia; d Department of Biosciences, College of Science, Swansea University, Swansea, Wales UK

**Keywords:** *Yersinia*, *Galleria*, adhesin, innate immunity, melanogenesis, damage response framework

## Abstract

Adhesins facilitate bacterial colonization and invasion of host tissues and are considered virulence factors, but their impact on immune-mediated damage as a driver of pathogenesis remains unclear. *Yersinia pseudotuberculosis* encodes for a multivalent adhesion molecule (MAM), a mammalian cell entry (MCE) family protein and adhesin. MAMs are widespread in Gram-negative bacteria and enable enteric bacteria to colonize epithelial tissues. Their role in bacterial interactions with the host innate immune system and contribution to pathogenicity remains unclear. Here, we investigated how*Y. pseudotuberculosis* MAM contributes to pathogenesis during infection of the *Galleria mellonella* insect model. We show that *Y. pseudotuberculosis* MAM is required for efficient bacterial binding and uptake by hemocytes, the host phagocytes. *Y. pseudotuberculosis* interactions with insect and mammalian phagocytes are determined by bacterial and host factors. Loss of MAM, and deficient microbe–phagocyte interaction, increased pathogenesis in *G. mellonella*. Diminished phagocyte association also led to increased bacterial clearance. Furthermore, *Y. pseudotuberculosis* that failed to engage phagocytes hyperactivated humoral immune responses, most notably melanin production. Despite clearing the pathogen, excessive melanization also increased phagocyte death and host mortality. Our findings provide a basis for further studies investigating how microbe- and host-factors integrate to drive pathogenesis in a tractable experimental system.

## Introduction

Understanding the functional role of microbial- and phagocyte surface components mediating host–pathogen interactions is essential to our ability to predict the overall outcome of host–pathogen interactions. Depending on the host’s ability to mount an appropriate immune response, the outcome may range from pathogen recognition and clearance (functional immunity), to immune evasion (failed immunity), or sepsis (hyperactivation of immunity). Both hypo- and hyperactivation of immune responses are detrimental to the host, and as such, pathogenicity is a function of both microbial and host factors – a concept termed the “damage response framework” [1,2].

Bacterial adhesins are key mediators of host–pathogen interactions; Pathogenic bacteria must adhere to host cells to deliver virulence factors that enable them to manipulate host signalling mechanisms and thus colonize and replicate within the host [3]. *Yersinia pseudotuberculosis* is a zoonotic, enteric pathogen that is transmitted via contaminated food and water. *Y. pseudotuberculosis* can attach to and invade host professional phagocytes (e.g. dendritic cells, macrophages), and it is by this intracellular route that it can survive, replicate, and disseminate *in vivo* [4–6]. One of the adhesins found on the surface of *Y. pseudotuberculosis*is the multivalent adhesion molecule (MAM), a mammalian cell entry (MCE) family protein (protein accession ACA68070.1, locus tag YPK_1779). As previously reported, *Y. pseudotuberculosis* MAM mediates bacterial association with epithelial cells, while a *Y. pseudotuberculosis* mutant lacking MAM exhibits diminished adherence and cytotoxicity toward epithelial cells [7]. The MAM protein sequence is highly conserved amongst *Yersinia spp*. (Figure S1), with *Yersinia pestis* MAM being the most closely related to *Y. pseudotuberculosis* MAM (Figure S2). Unlike other *Yersinia* adhesins (e.g. YadA, invasin, Ail), MAM homologs are present in a wide range of Gram-negative bacteria, although their ligand-binding specificities vary between species [3,8,9].

While prior work has characterized the role of MAM in bacterial interactions with epithelial cells in detail [7,10], its contribution to microbe–phagocyte interactions remains unclear. To understand the role of MAM during *Y. pseudotuberculosis* infection in a physiologically relevant model organism, we used insect larvae from the Greater wax moth, *Galleria mellonella*. The *Galleria* model system is relatively cost and labour effective, well adapted to growth at 37°C, and is free of ethical restrictions [11–13]. Injection of bacteria into the larval hemocoel (body cavity) is commonly used to study microbial interactions with cellular and humoral innate immune responses. Like most invertebrates, *G. mellonella’s* innate immune response is mechanistically similar to the innate immune system of vertebrates [14]. This immune response consists mainly of cellular and humoral components, with the cellular component made up of hemocytes. Hemocytes play a critical role in pathogen phagocytosis, encapsulation, nodulation, degranulation, etosis, and hemostasis events [15–17]. The humoral defenses are comprised of the soluble immune factors of the hemolymph, e.g. opsonins and antimicrobial peptides [18]. The most visible immune response is the activation of the pro-phenoloxidase cascade [19]. Briefly, microbes are detected by hemocyte subtypes, leading to the release of phenoloxidases (POs) from oenocytoids (crystal cells) that convert (mono- and di-) phenolic compounds into melanin precursors. Ultimately, the antimicrobial pigment melanin is produced and deposited on the microbe surface either to immobilize the invaders or used to coatdeveloping clots, resulting in darkening of the larval integument [20,21].

Herein, we show that *Y. pseudotuberculosis* MAM adhesin impacts pathogenesis in two ways: First, MAM facilitates bacterial binding and uptake by host phagocytes and in its absence, bacterial clearance is enhanced. Second, loss of bacteria – phagocyte interactions hyperactivates humoral immune responses, which is detrimental to the host and increases overall pathogenicity, despite lowering the bacterial burden. We also directly compare the binding and uptake capacity of insect and mammalian professional phagocytes and demonstrate that equivalent bacterial and phagocyte surface components mediate both processes in this experimental system. Together, these data provide a basis for further studies investigating how microbe- and host-factors integrate to drive pathogenesis in a tractable experimental system.

## Materials and methods

### Bacterial strains and growth conditions

The *Yersinia pseudotuberculosis* wild-type strain used was YP126 (YPIII, pYV^+^) [22]. An isogenic MAM deletion strain of YP126 was generated as described previously [7,23]. The deleted locus is annotated as the mammalian cell entry-related domain protein (GenBank: ACA68070.1, locus tag YPK_1779). Briefly, the deletion strain was generated by amplifying regions 1 kb upstream and downstream of the MAM coding sequence (Figure S3) using PCR with the following primers: CAGTAGATCTGAACCCCTGATCAGTATTCGC (−1kb fw)/CAGTACTAGTATGCATCCCAAATCAATCG (−1kb rev) and CAGTACTAGTATAATTCATCAGGGTGGTTCG (+1kb fw)/CAGTGTCGACCCGGTACCACTTTGAACACC (+1kb rev), respectively. PCR-amplified fragments were cloned into the suicide vector pDM4 [23] using BglII, SpeI, and SalI restriction sites. The resulting plasmid was transferred into *E. coli* SM10λpir cells by electroporation and the transformed strain was used as a donor strain for conjugal mating with YP126. After mating, positive clones were selected by re-plating on agar containing 25 µg/ml chloramphenicol and 10 µg/ml nalidixic acid. To cure the plasmid, individual colonies were re-streaked onto plates containing 15% sucrose. Colonies that grew on sucrose plates were patched onto plates containing 25 µg/ml chloramphenicol to confirm loss of chloramphenicol resistance. Finally, MAM deletion was confirmed by PCR and sequencing.

A plasmid for complementation of the ΔMAM strain was generated by amplification of MAM, including the endogenous MAM promoter region (see Supporting Information, SI) from YP126 genomic DNA using the following primers: CAGTCCATGGGTTAATCACGTTGCTGCTCAGT (fw)/CAGTAAGCTTTTAAGAACGTGGAATGGCTGT (rev). The resulting fragment was cloned into NcoI/HindIII restriction sites of a pBAD/myc-His plasmid that contained a kanamycin resistance cassette in place of the original ampicillin resistance marker, as described previously [7]. YP126 ΔMAM was transformed with the MAM complementation plasmid by electroporation to generate the strain YP126 ΔMAM+MAM (kan^R^). Since the endogenous MAM promoter is a sigma70 promoter, the complemented strain does not require arabinose to induce MAM expression. All bacterial strains were cultured in Lysogeny Broth (LB) at 37°C in an incubator shaking at 150–220 rpm for 16 h prior to further processing as described below. The complemented YP126 ΔMAM+MAM (kan^R^) strain was grown in LB that contained 50 µg/ml kanamycin. Based on spectrophotometric measurements, all three strains exhibited similar growth curve patterns (Figure S4).

### Galleria mellonella *larvae maintenance*

Final instar larvae of the greater wax moth, *G. mellonella*, were sourced from Livefoods Direct Ltd (UK) or Petco (US) and stored in wood shavings in the dark at 15°C. Healthy larvae weighing between 0.2 and 0.4 g were used in all experiments. Experimental manipulation of insects was approved by the University of Stirling’s Animal Welfare and Ethical Review Body (AWERB). Experimental manipulation of insects at McGovern Medical School was exempt from IACUC review as it constitutes an invertebrate model.

### Growth curves of Y. pseudotuberculosis strains

Wild-type *Y. pseudotuberculosis* YP126, ΔMAM, or ΔMAM+MAM were inoculated into LB-containing antibiotics (as described above) and incubated for 16 h at 37°C, shaking at 220 rpm. Cultures were diluted into fresh LB containing appropriate antibiotics and adjusted to an OD_600_ of 0.1. Samples were loaded onto 96-well plates, and OD_600_ was measured at every 30 min for 16.5 h using a FluoStar Omega plate reader. Plates were set to 37°C and to shake at 200 rpm for 5 min prior to each reading. Three different colonies per strain were used for the experiment.

### *G. mellonella* larval survival following *Y. pseudotuberculosis* challenge

Larvae weighing at least 0.2 g were injected with 20 µl of PBS or 20 µl of a bacterial suspension (8 × 10^7^ CFU/ml in PBS) of *Y. pseudotuberculosis* YP126 wild-type, ΔMAM, or ΔMAM+MAM complemented strains. Larvae were injected into the last right pro-leg using a 27.5-gauge hypodermic needle attached to a 20 µl pipette via a cutoff disposable pipette tip. Treated larvae (*n* = 27 per condition over 3 independent experiments) were incubated at 37°C and scored for mortality, melanization, and activity every 24 h for 7 days according to the health index scoring scheme developed by Loh et al [24].

### Impact of *Y. pseudotuberculosis* infection on G. mellonella larval melanization response

Larvae that weighed at least 0.2 g were injected with 20 µl of either PBS or a bacterial suspension (8 × 10^7^ CFU/ml in PBS) of wild-type *Y. pseudotuberculosis* YP126, ΔMAM, or ΔMAM+MAM. Following injection, larvae were incubated for 3 h at 37°C. Two representative larvae per condition were randomly selected and transferred to a 6-well plate for visual assessment of melanization. For quantitation of melanization, hemolymph was extracted from larvae through an incision behind the head. Approximately 20 µl hemolymph per larva was added to 20 µl ice-cold PBS containing 0.37% β-mercaptoethanol and kept on ice to stop further increases in melanin concentration. Samples were transferred to a 96-well U-bottom plate and absorbance at 405 nm was measured using a FluoStar Omega plate reader. Sample size included 10 larvae per condition across two independent experiments.

### Effect of synthetic melanin on *G. mellonella* larval infection with *Y. pseudotuberculosis*

To assess toxicity due to the melanization response, a stock of synthetic melanin was resuspended in 0.1 M NaOH at a concentration of 2 mg/ml. Larvae (*n* = 5 per treatment) were inoculated with different doses of synthetic melanin that ranged from 2 ng to 20 µg and incubated at 37°C. Larvae survival was scored every 24 h for 10 days after inoculation.

To study the putative protective effect of melanin during infection, larvae were inoculated with 20 μg melanin or left untreated and incubated at 37°C for 3 h. Then, larvae were injected with 20 µl of a bacterial suspension (8 × 10^7^ CFU/ml in PBS) of either the wild-type *Y. pseudotuberculosis* or the ΔMAM strain and incubated for an additional 72 h at 37°C. Larvae survival was scored at 72 h following bacterial inoculation using a total of 22 larvae per condition over four independent experiments.

### Hemocyte viability and bacterial burden following *G. mellonella* larvae infection with *Y. pseudotuberculosis*

Larvae that weighed at least 0.2 g (range 0.2–0.28 g) were injected with 20 µl PBS or a bacterial suspension (8 × 10^7^ CFU/ml in PBS) of wild-type *Y. pseudotuberculosis* YP126, ΔMAM, or ΔMAM. Following injections, larvae were incubated for 20 h at 37°C. To determine hemocyte viability, hemolymph was extracted from live larvae via an incision behind the head. Samples were harvested into 40 µl of ice-cold PBS that contained 0.37% β-mercaptoethanol and kept on ice until quantification. Total sample volumes were determined using a pipette. Trypan Blue was added to 60 µl of sample and adjusted to a final concentration of 0.02% (w/v). Viable (unstained) hemocytes were counted using a hemocytometer. The bacterial burden of *Yersinia* in hemolymph extracts was determined using serial dilutions of hemolymph in PBS. Dilutions were spotted onto LB agar plates that contained 50 µg/ml erythromycin.

Following exsanguination, larval tissues were cut into pieces using a sterile scalpel and samples were transferred to 2 ml tubes containing 1 ml PBS and 100 µm glass beads. Tissues were homogenized using a vortex mixer at maximum speed for 5 min. To remove glass beads, the resulting suspension was centrifuged at 300 × *g* for 10 min and serial dilutions of the supernatant were spotted onto LB agar plates supplemented with 50 µg/ml erythromycin. CFUs were enumerated following 20 h incubation at 37°C. No CFUs were detected on plates spotted with samples extracted from larvae injected with PBS alone or spotted with buffer as a control. Five live larvae were used for each experiment.

### *Y. pseudotuberculosis* attachment to *G. mellonella* hemocytes and melanization response ex vivo

*Y. pseudotuberculosis* strains were grown at 37°C, washed and resuspended in PBS to 8 × 10^7^ CFU/ml and stained with FM 4–64FX (25 µg/ml) for 30 min at 22°C. Samples were protected from light in order to prevent fluorophore bleaching. Bacteria were washed three times with PBS to remove excess stain and resuspended in DMEM to 8 × 10^7^ CFU/ml. Hemolymph was extracted from larvae via an incision behind the head and harvested into 20 µl of ice-cold PBS per larva. Larvae too small for previous injection experiments were used for this experiment (weight range 0.12 g-0.19 g). Hemolymph from a total of 15 larvae was pooled, resulting in 600 µl sample. Viable hemocytes were counted using a hemocytometer, then diluted into pre-warmed DMEM or DMEM that contained wild-type *Y. pseudotuberculosis* YP126, ΔMAM, or ΔMAM+MAM strains. Hemocyte suspensions were adjusted to achieve a density of 150,000 hemocytes/ml and a bacterial MOI (multiplicity of infection) of 200 bacteria/hemocyte. Following resuspension, 1 ml volumes of each sample were loaded into a glass-bottom 24-well plate.

To study the impact of RGD peptide on bacterial association with hemocytes, RGD peptide (≥97% purity, Sigma A8052), was reconstituted in ultra-pure dH_2_O to a concentration of 1 mM, and stocks were stored at −20°C. Prior to addition of *Y. pseudotuberculosis* strains as described above, uninfected hemocytes were treated with RGD peptide at the time of plating and incubated for 30 min at 37°C (20 µM RGD, final concentration). For the duration of infection, plates containing hemocytes and bacteria were incubated for 1 h at 37°C in 5% CO_2_. Plates were protected from light exposure during this incubation and in all subsequent incubation steps. Following infection, media was discarded, wells were washed with PBS, and cells were fixed with 3.7% formaldehyde in PBS for 20 min at 22°C. Cells were subjected to PBS washes, permeabilized with 1% Triton X-100 in PBS for 20 min at 22°C and washed with PBS again. Finally, cells were stained with 0.3 µM DAPI and 0.2 mg/ml Concanavalin A-Alexa Fluor 488 Conjugate in PBS for 20 min at 22°C. Wells were washed 3x with PBS and kept in 1 ml PBS for imaging.

Samples were visualized on an Olympus IX83 inverted microscope fitted with a FV3000 laser scanning confocal system and a UCPLFLN20X objective. Images were processed using Olympus CellSens Dimension software/deconvolution package and ImageJ. Data represent a sample population of ≥ 100 host cells that were analyzed to quantify bacterial association with hemocytes, across a minimum of four independent experiments. Melanin production was quantified based on bright field images as total area covered by melanin per field and average area covered by an individual melanin cluster (on an average of 60 clusters), across a minimum of four independent experiments.

### Impact of RGD peptide on *G. mellonella* larval infection by *Y. pseudotuberculosis*

Larvae were injected with 20 µl of a 20 μM RGD peptide solution or 20 µl PBS as a control into the last right pro-leg and incubated for 120 min at 37°C. Larvae were then injected with 20 μl of a bacterial suspension (8 × 10^7^ CFU/ml in PBS) of wild-type *Y. pseudotuberculosis* YP126, ΔMAM, or ΔMAM complemented strains, or PBS alone, into the last left pro-leg. Larvae were incubated at 37°C and scored for mortality, melanization, and activity every 24 h for 3 days using the health index scoring system by Loh et al. [24]. A total of 12 larvae per condition were scored.

### Attachment of *Y. pseudotuberculosis* to mammalian macrophages

RAW264.7 murine macrophages (cell line TIB-71, American Type Culture Collection, Manassas, VA) were maintained in DMEM supplemented with 1% L-glutamine, 1% Penicillin/Streptomycin, and 10% FBS at 37°C under 5% CO_2_. 24 h prior to the experiment, macrophages were harvested by scraping cells into DMEM and adjusted to a concentration of 150,000 cells/ml). To seed macrophages, 0.5 ml volumes of the cell suspension was transferred into each well of a 24-well glass bottom plate.

One hour prior to infection, macrophages were incubated with 20 µM of RGD peptide (20 µl/ml in pure DMEM) or an equivalent volume of PBS in pure DMEM (1 h, 37°C). For the attachment assay, *Y. pseudotuberculosis* strains were stained with FM 4–64FX as described above, resuspended in pure DMEM (MOI = 200), and added to macrophages. An MOI of 200 was used to keep the experimental conditions equivalent to those used for hemocyte infections. Samples were incubated for 1 hour at 37°C under 5% CO_2_. Samples were further processed for fluorescence microscopy, and images were acquired as described above (for hemocyte attachment assays). To quantify bacterial attachment, data from a minimum of 200 host cells across four independent experiments were analyzed.

### *Y. pseudotuberculosis* uptake by phagocytes

To quantify the burden of intracellular bacteria within *G. mellonella* hemocytes and RAW264.7 murine macrophages, respectively, cells were plated as described above for attachment assays. Bacteria were grown, pre-stained with FM 4–64FX, and resuspended in DMEM without supplements to infect phagocytes at 37°C. At 30 min post infection, media was removed, cells were washed, DMEM containing 250 µg/ml gentamicin and 150 µg/ml spectinomycin was added, and plates were incubated at 37°C for a further 1.5 h. Following incubation, cells were fixed and stained for imaging as described above.

### Statistical analyses

For survival analyses of *G. mellonella* larvae following *Y. pseudotuberculosis* infection, a log-rank (Mantel-Cox) and Fisher’s test was used. For all other statistical analyses, mean activity from larvae challenged with wild-type *Y. pseudotuberculosis* YP126, ΔMAM, or ΔMAM+MAM strains was compared using one- or two- way ANOVA and Dunnett’s multiple comparisons tests with (****) p ≤ 0.0001, (***) p ≤ 0.001, (**) p ≤ 0.01, (*) p ≤ 0.05, (ns, not significant) p ≥ 0.05. Data analyses were performed using GraphPad Prism 8.4.3 (GraphPad Software, San Diego, California USA).

## Results

### Deletion of the *Y. pseudotuberculosis* adhesin MAM increases pathogenicity in the Galleria mellonella larval infection model

**Figure 1.**
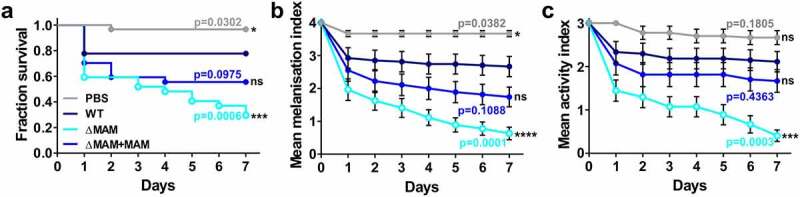
**Deletion of the *Yersinia pseudotuberculosis* adhesin MAM increases pathogenicity in the *Galleria mellonella* larval infection model**. Larvae were injected with 20 µl each of PBS, or a suspension of PBS containing 8 × 10^7^ CFU/ml of *Y. pseudotuberculosis* WT, ΔMAM, or ΔMAM+MAM complemented strains. 27 larvae/condition) were scored for survival (a), melanization health score index (b), and activity health score index (c) every 24 h for 7 days. Data in (b) and (c) are means ± s.e.m. Significance in survival compared to larvae infected with WT *Y. pseudotuberculosis* was determined using a log-rank (Mantel-Cox) and Fisher’s test. Significance between mean melanization scores and mean activity scores compared to larvae infected with WT YP was determined using two-way ANOVA and a Dunnett’s multiple comparisons test

First, we determined the relative virulence of the wild-type and mutant *Y. pseudotuberculosis* strains following infection of *G. mellonella* by quantifying larval survival, melanization, and activity health index scores [24]. In agreement with previous reports, larval injection of *Y. pseudotuberculosis* significantly decreased host survival (Figure 1(a)), increased melanization (Figure 1(b)), and decreased larval activity (Figure 1(c)) compared to PBS-injected larvae [25].

To test whether expression of the MAM adhesin affects *Y. pseudotuberculosis* virulence, we also injected larvae with a *Y. pseudotuberculosis* MAM deletion strain (ΔMAM), or the MAM deletion strain carrying a MAM complementation plasmid (ΔMAM+MAM). At 37°C, the growth rates of wild-type, MAM-deficient, and MAM-complemented *Y. pseudotuberculosis* strains were not significantly different (Figure S4). Compared to wild-type *Y. pseudotuberculosis*, infection with a MAM-deficient strain (ΔMAM) elicited increased overall mortality in *G. mellonella* larvae (Figure 1(a)), increased melanization (Figure 1(b)), and decreased larval activity (Figure 1(c)). Larval infection by the complemented MAM deletion strain (ΔMAM+MAM) elicited similar patterns of host response to WT strain infection (Figure 1(a**–**c)).

### Deletion of *Y. pseudotuberculosis* MAM adhesin decreases both pathogen- and hemocyte viability following *G. mellonella* infection

*G. mellonella* larvae contain several hemocyte subtypes that play key roles both in cell-dependent and humoral immune responses to invading pathogens. While plasmatocytes and granular cells adhere to and remove pathogens via phagocytosis, oenocytes are non-phagocytic cells that release components of the phenoloxidase cascade and contribute to melanization [18].

**Figure 2.**
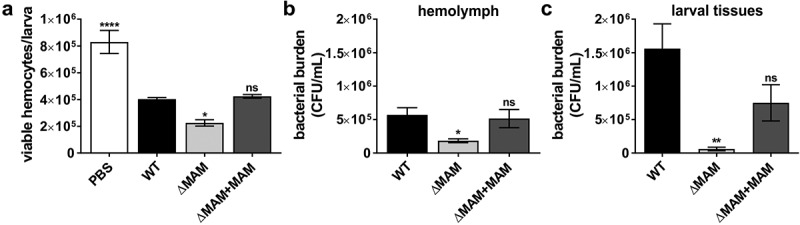
**Deletion of *Y. pseudotuberculosis* MAM adhesin decreases both pathogen- and hemocyte viability following *G. mellonella* infection**. Larvae were injected with PBS, or a suspension of PBS containing 8 × 10^7^ CFU/ml of wild-type *Y. pseudotuberculosis* YP126, ΔMAM, or ΔMAM containing a MAM complementation plasmid (ΔMAM+MAM), and incubated for 20 h at 37°C. (a) Hemolymph was extracted and viable hemocytes were quantified following trypan blue staining. (b) Hemolymph was extracted and (c) remaining larval tissues homogenized, and bacterial burdens were determined by dilution plating on erythromycin agar. Values are means ± s.e.m. from 5 larvae/condition. Statistical significance compared to larvae infected with wild-type YP was determined by one-way ANOVA, and a Dunnett’s multiple comparisons test. (****) p ≤ 0.0001, (**) p ≤ 0.01, (*) p ≤ 0.05, (ns, not significant) p ≥ 0.05

To further investigate how loss of MAM adhesin led to increased larval mortality, we examined hemocyte viability and bacterial burden both in hemolymph and larval tissues following infection. Insect larvae were infected via 20 µl injections of wild-type *Y. pseudotuberculosis*, ΔMAM, or ΔMAM+MAM strains (8x10^7^ CFU/ml) and incubated for 20 h at 37°C. The number of viable hemocytes isolated from larval hemolymph significantly decreased in response to *Y. pseudotuberculosis* infection by all strains when compared to the number of viable hemocytes contained in PBS-injected larvae (Figure 2(a)). Larvae infected with *Y. pseudotuberculosis* ΔMAM contained hemolymph with significantly depleted levels of viable hemocytes compared to that of wild-type- or ΔMAM+MAM-infected larvae (Figure 2(a)).

Next, we examined the burden (i.e. the number of viable bacteria) of *Y. pseudotuberculosis* in either larval hemolymph or solid tissue at 20 h post-infection. To distinguish *Y. pseudotuberculosis* from other microbes, hemolymph and tissue homogenates used for burden determination were plated on agar containing erythromycin, which suppressed all background microbial growth in samples extracted from the negative control (PBS-injected larvae). The bacterial burdens in larvae infected with *Y. pseudotuberculosis* ΔMAM were significantly lower than in larvae infected with either wild-type bacteria or ΔMAM+MAM complemented strains. This was true for both hemolymph (Figure 2(b)) and solid tissue samples (Figure 2(c)).

### Deletion of *Y. pseudotuberculosis* MAM adhesin exacerbates the early immune response of *G. mellonella* to infection

**Figure 3.**
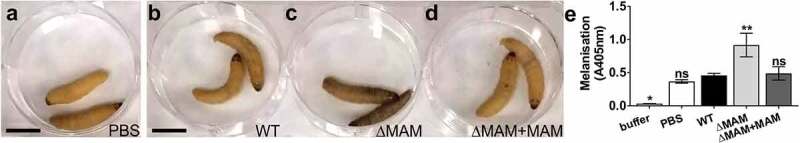
**Deletion of *Y. pseudotuberculosis* MAM adhesin exacerbates the early melanization response of *G. mellonella* to infection**. Larvae were injected with PBS (a), or a suspension of PBS containing 8 × 10^7^ CFU/ml of wild-type *Y. pseudotuberculosis* YP126 (b), ΔMAM (c) or ΔMAM containing a MAM complementation plasmid (d). Larvae were imaged following 3 h incubation at 37°C. Scale bar, 1 cm. (e) Hemolymph was extracted from larvae treated as in A-D, and melanin content quantitated by measuring absorbance at 405 nm. Data are means ± s.e.m. (10 larvae/condition); Statistical significance compared to larvae infected with wild-type YP was determined by one-way ANOVA, and a Dunnett’s multiple comparisons test. (**) p ≤ 0.01, (*) p ≤ 0.05, (ns, not significant) p ≥ 0.05

To further characterize the early larval immune response to *Y. pseudotuberculosis* infection, we visually assessed and quantitated melanization 3 h post-infection (Figure 3). No melanization was observed in PBS-injected larvae (Figure 3(a)), while larvae infected with wild-type *Y. pseudotuberculosis* or complemented MAM deletion strain (ΔMAM+MAM) either did not develop melanization or showed slight (localized) pigmentation (Figure 3(b,d)). In contrast, pigmentation of larvae infected with the MAM deletion strain (ΔMAM) appeared significantly increased at 3 h post-infection (Figure 3(c)). For objective quantification of melanization, we measured melanin levels contained in hemolymph isolated from larvae 3 h post-infection (Figure 3(e)). Consistent with the visual appearance of the integument, the hemolymph isolated from *Y. pseudotuberculosis* ΔMAM-infected larvae contained significantly increased melanin levels compared to the hemolymph of larvae infected with wild-type *Y. pseudotuberculosis* at 37°C (Figure 3(e)). Melanin levels in the hemolymph of larvae injected with wild-type bacteria or the ΔMAM+MAM strain were not significantly increased compared to levels in PBS-injected larvae (Figure 3(e)).

### Deletion of *Y. pseudotuberculosis* MAM adhesin decreases bacterial binding and uptake by hemocytes and exacerbates melanization

**Figure 4.**
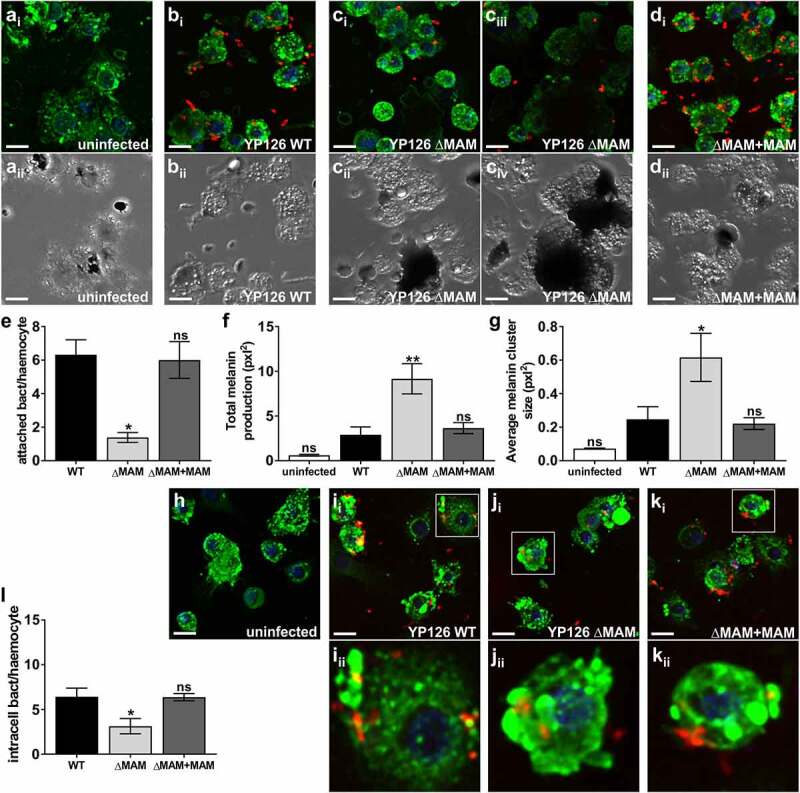
**Deletion of *Y. pseudotuberculosis* MAM adhesin decreases bacterial binding and uptake by hemocytes and exacerbates melanization**. For bacterial association assays (a-g), extracted hemocytes were PBS-treated and left uninfected (a), or infected with wild-type *Y. pseudotuberculosis* (b), ΔMAM (c), or ΔMAM complemented with a MAM containing plasmid (d) at an MOI of 200 for 1 hr at 37°C. For bacterial uptake assays (h-l), PBS-treated hemocytes were left uninfected (h), or infected with WT (i), ΔMAM (j), or ΔMAM+MAM (k) at an MOI of 200 for 30 min, and intracellular bacteria visualized following a gentamicin protection assay for 1.5 h at 37°C. Images are MIPs of representative z-stacks; scale bars, 10 µm. Ai-Di & H-K: *Yersinia* (FM 4–64FX, red), DNA (DAPI, blue), hemocyte surface (ConA, green). Aii-Dii: bright field, melanin visible as black clumps. (e) Image-based quantification of hemocyte-associated bacteria; Data from ≥100 host cells (n = 4 experiments) were quantified. (f) Total melanin was quantified as total pixel area covered by melanin/imaged field. (g) Average melanin cluster size was quantified as average pixel area covered by melanin clusters/imaged field. For F and G, an average of 60 clusters (n = 4 experiments) were quantified. (l) Image-based quantification of intracellular bacteria/hemocyte; Data from ≥110 host cells (n = 6 experiments) were quantified. Graphs show means ± s.e.m. Statistical significance compared to wild-type infection was determined by one-way ANOVA, and a Dunnett’s multiple comparisons test. (**) p ≤ 0.01, (*) p ≤ 0.05, (ns, not significant) p ≥ 0.05

Although the *Y. pseudotuberculosis* MAM deletion strain appeared to be hypervirulent in larvae (Figure 1), its bacterial burden following infection was lower than that of the wild-type strain (Figure 2). The fact that virulence and burden do not correlate suggests that mortality may not be purely pathogen-driven. Additionally, infection of larvae with *Y. pseudotuberculosis* ΔMAM decreased hemocyte viability (Figure 2), while increasing melanization early during infection (Figures 1(b) and 3), suggesting differences in host immune response toward wild-type and ΔMAM strains may play a role in pathogenesis. To test this hypothesis, we isolated hemolymph from unchallenged larvae, plated the hemocytes contained therein, then incubated host cells with fluorescent *Y. pseudotuberculosis* at an MOI of 200 for 1 h at 37°C. This allowed us to visualize and quantify the ability of *Y. pseudotuberculosis* to attach to and be internalized by hemocytes using the resultant, fluorescent z-stacks Figure 4(ai-di,h-k). Compared to samples that included wild-type or MAM-complemented *Y. pseudotuberculosis* strains, the number of hemocyte-associated bacterial cells was significantly reduced in samples containing the *Y. pseudotuberculosis* MAM deletion strain (Figure 4(ai–di,e)). Concurrently, the host’s melanization response to extracellular bacteria was assessed using bright field images of the same hemocytes observed in Figure 4(ai–di) (Figure 4(aii–dii)). To do so, the area of melanin clusters in infected hemocytes was measured (Figure 4(aii–dii)). Both the overall amount of melanin produced (Figure 4(f)), and the average melanin cluster size (Figure 4(g)) increased in infected hemocyte samples (Figure 4(bii–dii)), compared to uninfected hemocytes (Figure 4(aii)). Overall, hemocytes infected with the *Y. pseudotuberculosis* ΔMAM strain (Figure 4(cii)) produced more total melanin and larger melanin clusters compared to hemocytes infected with wild-type *Y. pseudotuberculosis* (Figure 4(bii)) or ΔMAM+MAM complemented strains (Figure 4(dii)).

Finally, we investigated if the decreased association of *Y. pseudotuberculosis* ΔMAM bacteria with *G. mellonella* hemocytes would also lead to decreased bacterial uptake. To assess bacterial uptake, we used the same experimental approach as above but treated infected hemocytes with gentamycin and spectinomycin 30 min post-infection to kill extracellular bacteria. The infection continued for an additional 90 min in the presence of antibiotics before imaging (Figure 4(h**–**k)) and quantification (Figure 4(l)) of intracellular *Y. pseudotuberculosis*. While wild-type *Y. pseudotuberculosis* (Figure 4(i)) and complemented deletion (ΔMAM+MAM) strains (Figure 4(k)) were internalized by hemocytes at similar levels, intracellular levels of ΔMAM bacteria (Figure 4(j)) were significantly reduced in comparison (Figure 4(l)).

### Inhibition of integrin-mediated association of *Y. pseudotuberculosis* with hemocytes exacerbates melanization and pathogenicity

Together, our results suggest that the adhesin MAM plays a significant role in mediating *Y. pseudotuberculosis* binding and uptake by hemocytes. However, *Y. pseudotuberculosis* expresses additional adhesins that have been shown to promote bacterial association with mammalian cells, including invasin and YadA. Invasin directly binds α5β1 integrin [26] and mediates invasion of the intestinal epithelium via M cells [27], while YadA binds β1 integrin indirectly via fibronectin, and mediates binding and invasion of phagocytes. YadA is maximally expressed at 37°C, under conditions where invasin is repressed [28]. Arg-Gly-Asp (RGD) is a well-characterized tripeptide that binds to integrins [29] and, if added to cells, blocks integrin-mediated processes such as phagocytosis both in vertebrates and invertebrates [30].

**Figure 5.**
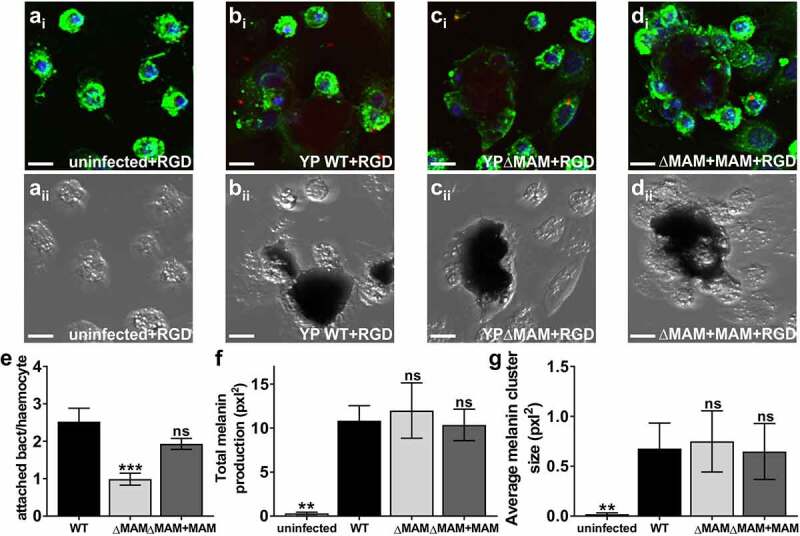
**Inhibition of integrin-mediated association of *Y. pseudotuberculosis* with hemocytes exacerbates melanization**. Extracted hemocytes were treated with 20 μM RGD peptide for 30 min and either left uninfected (a), or infected with wild-type *Y. pseudotuberculosis* (b), ΔMAM (c), or ΔMAM complemented with a MAM containing plasmid (d) at an MOI of 200 for 1 hr at 37°C. Images are MIPs of representative z-stacks; scale bars, 10 µm. Ai-Di: *Yersinia* (FM 4–64FX, red), DNA (DAPI, blue), hemocyte surface (ConA, green). Aii-Dii: bright field, melanin visible as black clumps. (e) Image-based quantification of hemocyte-associated bacteria; Data from ≥200 host cells (n = 6 experiments) were quantified. (f) Total melanin was quantified as total pixel area covered by melanin/imaged field. (g) Average melanin cluster size was quantified as average pixel area covered by melanin clusters per imaged field. For F and G, an average of 40 clusters (n = 6 experiments) were quantified. Graphs show means ± s.e.m. Statistical significance was determined by one-way ANOVA, and a Dunnett’s multiple comparisons test. (***) p ≤ 0.001, (**) p ≤ 0.01, (ns, not significant) p ≥ 0.05

To investigate whether the above-mentioned *Y. pseudotuberculosis* adhesins contribute to bacterial binding and uptake by hemocytes, we blocked integrins with RGD and compared association of wild-type and MAM deletion strains with hemocytes *ex vivo*. Hemocytes were isolated from unchallenged larvae, plated, and treated with 20 μM RGD peptide to block integrins. RGD-treated hemocytes were challenged with fluorescently labeled *Y. pseudotuberculosis* and both bacterial association (Figure 5(Ai–Di)) and hemocyte melanization response (Figure 5(Aii–Dii)) were imaged and quantified (Figure 5(e–g)) as above. The number of attached bacterial cells significantly decreased in samples that included wild-type (Figure 5(Bi)) or ΔMAM+MAM (Figure 5(Di)) *Y. pseudotuberculosis* cells, but not the ΔMAM cells (Figure 5(Ci)), when hemocytes were pretreated with RGD peptide (compared to untreated hemocytes, Figure S5). The decreased bacterial attachment of wild-type and ΔMAM+MAM strains to RGD-treated hemocytes was associated with significant increases in total melanin production (Figure 5(f)) and melanin cluster size (Figure 5(g)). Overall, the melanization response of RGD-treated hemocytes to all three *Y. pseudotuberculosis* strains was similarly high (Figure 5(Bii–Dii)), while RGD treatment alone (Figure 5(Aii, F, G)) did not increase melanization compared to PBS treatment (Figure 4(Aii, F, G)).

**Figure 6.**
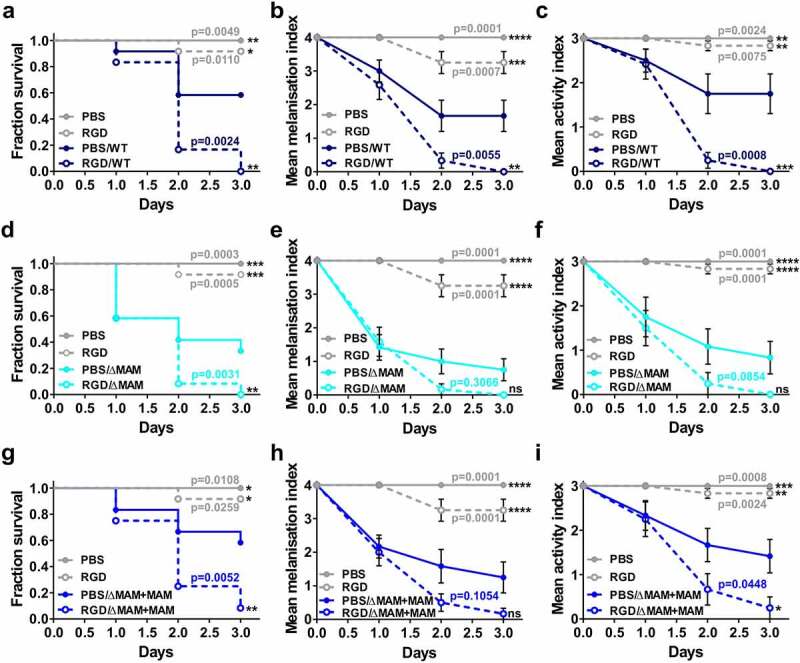
**Larval treatment with integrin blocking peptide RGD exacerbates *Y. pseudotuberculosis* infection**. Larvae were injected with 20 μl of 20 μM RGD peptide solution or 20 μl PBS and incubated at 37 °C for 120 min, and then injected with 20 μl of a suspension of PBS containing 8 × 10^7^ CFU/ml of *Y. pseudotuberculosis* WT **(a-c**, dark blue), ΔMAM **(d-f**, light blue), or ΔMAM+MAM complemented **(g-i**, middle blue) strains. 12 larvae/condition were scored for survival **(a, d, g)**, melanization health score index **(b, e, h)**, and activity health score index **(c, f, i)** every 24 h for 3 days. Melanization and activity indices are means ± s.e.m. For each strain, significance in larval survival compared to larvae infected with *Y. pseudotuberculosis* following PBS injection was determined using a log-rank (Mantel-Cox) and Fisher’s test. Significance in mean melanization scores and mean activity scores compared to larvae infected with *Y. pseudotuberculosis* following PBS injection was determined using two-way ANOVA and a Dunnett’s multiple comparisons test. (****) p ≤ 0.0001, (***) p ≤ 0.001, (**) p ≤ 0.01, (*) p ≤ 0.05, (ns, not significant) p ≥ 0.05

Next, we used the larval injection model to test how integrin inhibition via RGD treatment would affect *Y. pseudotuberculosis* infection *in vivo*. Larval injections of 20 μM RGD peptide did not affect host survival, melanization, or activity compared to injections of PBS alone (Figure 6). This suggests that treatment with RGD peptide alone does not negatively impact larval physiology. However, treatment of larvae with RGD peptide exacerbated subsequent infections with wild-type or ΔMAM+MAM *Y. pseudotuberculosis* strains. Significant increases in mortality (Figure 6(a,g)) and melanization (Figure 6(b,h)) and decreased larval activity (Figure 6(c,i)) were observed compared to PBS-treated, infected larvae. While RGD treatment also increased mortality of larvae challenged with *Y. pseudotuberculosis* ΔMAM (Figure 6(d)), treatment did not significantly impact melanization (Figure 6(e)) or larval activity (Figure 6(f)) following ΔMAM infection. Together, these data suggest that integrins play an important role in mediating *Y. pseudotuberculosis* – hemocyte interactions during *G. mellonella* infection, and that inhibition of *Y. pseudotuberculosis –* integrin association during infection *in vivo* increases pathogenicity.

### Injection of *G. mellonella* with preformed melanin protects against hyperactivation of melanogenesis

**Figure 7.**
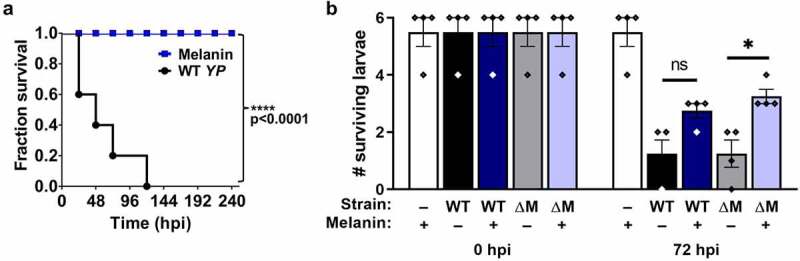
**Injection of *G. mellonella* with preformed melanin protects against hyperactivation of melanogenesis**. (a) Larvae were inoculated with 20 μg of synthetic melanin (blue) or a bacterial suspension (8 × 10^7^ CFU/ml in PBS) of wild-type *Y. pseudotuberculosis* (black), and larval survival scored every 24 h for 240 h (5 larvae/condition, 1 experiment); Significance in larval survival compared to larvae infected with *Y. pseudotuberculosis* was determined using a log-rank (Mantel-Cox) and Fisher’s test. (****) p ≤ 0.0001. (b) Larvae were injected with 20 μg melanin pigment (+), or PBS as a control (-), and incubated for 3 h, followed by injection of PBS alone (-, white bars), or a bacterial suspension (8 × 10^7^ CFU/ml in PBS) of wild-type *Y. pseudotuberculosis* (WT, black/dark blue bars) or an isogenic MAM deletion strain (∆M, grey/light blue bars). Surviving larvae at 0 h or 72 h post-infection (hpi) are indicated (n = 22 larvae per condition over 4 independent experiments)

Melanin has been described to confer protective immunity to larvae by binding to the cell surface of invading bacteria and forming nodules/capsules containing pigment and aggregated pathogens [20]. However, the release of phenoloxidases (PO) in response to pathogen-associated molecular pattern (PAMP) recognition involves the lysis of PO-containing hemocytes and the production of reactive intermediates that are toxic toward *G. mellonella* [31]. Thus, hyperactivation of melanogenesis may potentially be deleterious for *G. mellonella*, which is in line with our data above. We tested whether injection of larvae with preformed melanin, which would bypass the harmful effects of melanin biogenesis, would protect against subsequent *Y. pseudotuberculosis* challenge (Figure 7). Injection of larvae with a dose of 20 μg exogenous melanin did not cause any larval mortality for up to 240 h post-injection (Figure 7(a)). As expected, *Y. pseudotuberculosis* injections performed on the same batch of larvae as a positive control caused mortality (Figure 7(a)). Larval injections of 20 μg melanin 3 h prior to pathogen challenge conferred partial protection against infection with MAM-deficient, but not wild-type *Y. pseudotuberculosis* (Figure 7(b)). These data suggest that the larval mortality we observe is largely due to hyperactivation of melanogenesis in the presence of a large bacterial burden in the hemocoel, and that injection of larvae with melanin pigment is protective in the context of *Y. pseudotuberculosis* ΔMAM infection, where it coats extracellular bacteria that fail to associate with hemocytes.

### *Y. pseudotuberculosis* MAM and phagocyte integrins mediate binding and uptake of bacteria by mammalian macrophages

**Figure 8.**
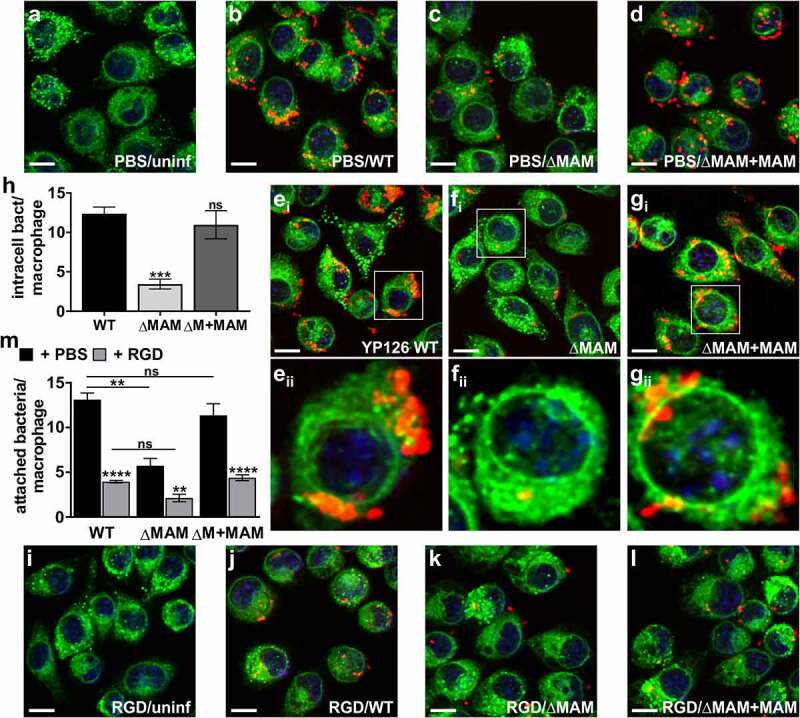
***Y. pseudotuberculosis* MAM and phagocyte integrins mediate binding and uptake of bacteria by mammalian macrophages**. For bacterial association assays, RAW264.7 murine macrophages were PBS-treated (a-d) or treated with 20 µM RGD peptide (i-l) for 1 h, and left uninfected (a, i), or infected with wild-type *Y. pseudotuberculosis* (b, j), ΔMAM (c, k), or ΔMAM+MAM (d, l) at an MOI of 200 for 1 hr at 37°C. For bacterial internalization assays (e-h), PBS-treated macrophages were infected with WT *Y. pseudotuberculosis* (e), ΔMAM (f), or ΔMAM+MAM (g) at an MOI of 200 for 30 min, and intracellular bacteria visualized following a gentamicin protection assay for 1.5 h at 37°C. All images are MIPs of representative z-stacks; scale bars, 10 µm. *Yersinia* (FM 4–64 FX, red), DNA (DAPI, blue), macrophage surface (ConA, green). (h) Image-based quantification of intracellular bacteria/macrophage; Data from ≥160 host cells (n = 4 experiments) were quantified. (m) Image-based quantification of macrophage-associated bacteria; Data from ≥200 host cells (n = 4 experiments) were quantified. Graphs show means ± s.e.m. For (h)and (m), statistical significance compared to wild-type infection was determined by one-way ANOVA, and a Dunnett’s multiple comparisons test. For M, for each strain, statistical significance of data between PBS- and RGD-treated macrophages was determined the same way. (****) p ≤ 0.0001, (***) p ≤ 0.001, (**) p ≤ 0.01, (ns, not significant) p ≥ 0.05

Although other adhesins have been implicated in *Y. pseudotuberculosis* association and invasion of mammalian macrophages [28,32], those studies have not tested the contribution of *Y. pseudotuberculosis* MAM. Additionally, the capacities for bacterial binding and uptake of mammalian and insect professional phagocytes have not been directly compared in prior studies. To address these gaps in knowledge, we imaged and quantified *Y. pseudotuberculosis* binding and uptake by RAW 264.7 mouse macrophages following treatment with either RGD peptide or PBS alone. We used an MOI of 200, to make the results directly comparable to those obtained for binding and uptake by hemocytes. For all three *Y. pseudotuberculosis* strains, bacterial association with PBS-treated mouse macrophages (Figure 8(a**–**d)) and uptake by mouse macrophages (Figure 8(e**–**h)) were significantly higher than binding and uptake by *G. mellonella* hemocytes (see Figure S6 for side-by-side comparison). For PBS-treated mouse macrophages infected with the *Y. pseudotuberculosis* MAM deletion strain (Figure 8(c)), the number of macrophage-associated bacteria was significantly reduced compared to macrophages infected with wild-type (Figure 8(b)) or MAM-complemented strains (Figure 8(d,m)). This defect in bacterial association (Figure 8(m)) was also reflected in the significant decrease in bacterial uptake by mouse macrophages (Figure 8(h)) infected with *Y. pseudotuberculosis* ΔMAM (Figure 8(f)) compared to wild-type- (Figure 8(e)) and ΔMAM+MAM-infected macrophages (Figure 8(g)). Pretreatment of mouse macrophages with 20 µM RGD peptide did not cause cell death or change cell morphology compared to PBS-treated cells (Figure 8(a,i)). RGD peptide-mediated inhibition of surface-expressed integrins on macrophages significantly reduced bacterial association for all three *Y. pseudotuberculosis* strains (Figure 8(j**–**m)). Taken together, these data suggest that binding and uptake of *Y. pseudotuberculosis* by insect and mammalian macrophages are driven by analogous bacterial and host molecular determinants that are found at the cell surface. However, initial attachment of *Y. pseudotuberculosis* to mammalian macrophages and consequent uptake efficiency is enhanced compared to bacterial binding and uptake by insect hemocytes.

## Discussion

Multivalent adhesion molecules (MAMs) are found in many Gram-negative bacteria, and *Yersinia* MAMs are highly conserved between different *Yersinia* spp. (Figures S1, S2). While previous work has characterized the receptor-binding specificity of MAMs and their role in epithelial colonization by enteric bacteria including *Vibrio parahaemolyticus* and *E. coli* [7,9], their contribution to interactions between bacteria and host innate immune responses remained unclear. *Y. pseudotuberculosis* commonly infects the host via the fecal-oral route, and successful infection relies on bacterial persistence and replication both in the blood and inside phagocytes [33]. The *G. mellonella* larval model lends itself to the study of aspects of bacterial pathogenesis that are driven by bacteria – innate immune interactions. Injection of bacteria into the larval hemocoel directly exposes them to the host’s humoral and phagocytic immune responses, which can be assessed both visually and quantitatively. *G. mellonella* larvae are well established as a non-vertebrate model of host–microbe interactions and are widely used to test the contribution of bacterial virulence factors to pathogenesis [34–36].

Prior studies of *Y. pseudotuberculosis* in *G. mellonella* reported time- and dose-dependent larval mortality in response to bacterial challenge and are in agreement with our data [25,37,38]. These studies reported decreased *Y. pseudotuberculosis* virulence toward larvae in the absence of bacterial factors required for oxidative stress and antimicrobial resistance, or intracellular persistence and replication [25,37,38]. However, the mutants studied in this context were deficient in bacterial persistence and did not affect bacterial binding or uptake by insect or mammalian phagocytes [25,39]. Although other studies reported that *Y. pseudotuberculosis* also elicits a humoral response in *G. mellonella*, including visual melanization and enhanced antimicrobial activity of the larval hemolymph [37,40], none fully characterized or quantified the link between bacterial phagocytosis, humoral responses, and host survival.

Hemocytes and monocytes, the primary mediators of cell-mediated innate immunity in invertebrates and mammals, respectively, are largely equivalent both functionally and in terms of the surface receptors and downstream signalling events involved in bacterial recognition and internalization [30,41]. Both cell types depend on β integrins for pathogen recognition and internalization. Exposure to β integrin-blocking antibodies or RGD peptide inhibits integrin-mediated bacterial uptake [30]. However, no prior studies have systematically compared pathogen binding and uptake efficiency of hemocytes and mammalian macrophages, nor have they considered the relative contributions of bacterial and host receptors to each system. Our data suggest that *Y. pseudotuberculosis* is bound and taken up by both insect hemocytes and mammalian macrophages, but the efficiency of both processes is approximately two-fold greater in macrophages than in hemocytes in the absence of prior activation and/or bacterial opsonization (Figures 4, 8, S6). Inhibition of integrins on hemocytes and macrophages by RGD peptide decreased wild-type *Y. pseudotuberculosis* attachment by approximately 3-fold for both cell types (Figures 5, 8, S5). These data demonstrate the importance of integrins to *Y. pseudotuberculosis* – phagocyte interactions for both insect and mammalian hosts.

Deletion of MAM decreased bacterial association with hemocytes and macrophages by approximately 5- and 2-fold, respectively. The decrease in initial bacterial attachment was also reflected in diminished uptake of the MAM-deficient strain by both cell types (approx. 2- and 4-fold, respectively). These data suggest that the *Y. pseudotuberculosis* adhesin MAM plays an important role in bacteria-phagocyte interactions in both insect and mammalian systems under the experimental conditions tested here. We show that in the absence of MAM, RGD peptide treatment further reduces bacterial association with phagocytes, although the decrease in ΔMAM adherence observed in response to RGD is small in hemocytes, likely due to the low absolute attachment under this condition (Figures 4, 5, S5). Taken together, our data suggest that MAM, along with other adhesins, mediate bacterial binding by insect and mammalian phagocytes and associates with the phagocyte surface via integrins and other host receptors. Previously, MAM has been shown to interact with fibronectin, so MAM’s association with phagocyte β integrins is likely to be indirect and mediated by fibronectin, as is the case for other *Y. pseudotuberculosis* adhesins [42,43]. Residual association of bacteria with macrophages in the absence of MAM and free β integrins may be mediated by alternative bacteria-host receptor interactions, which have also been observed in cultured mammalian cells and rodent models but as of yet have not been fully characterized [39,44].

As mentioned above, melanin production is an essential component of invertebrate innate immunity and response to microbial intrusion [19,20]. Depending on the host, melanization may be triggered in response to host cell death and/or PAMPs such as LPS and peptidoglycan [45]. Endogenous or exogenous signals lead to the release of soluble antimicrobial factors and POs via hemocyte rupture and, ultimately, melanization [45–47]. We quantitated both bacterial association and melanization of hemocytes in the same experiment and found a strong negative correlation between bacterial association/phagocytosis and melanization (Figures 4 and 5). Wild-type bacteria that were rapidly internalized caused no significant change in melanin production within the first hours post-infection (Figures 3 and 4) and very limited melanization even several days post-infection (Figure 1). In contrast, bacteria that failed to efficiently associate with phagocytes activated melanin production, causing the formation of cellular aggregates that contained bacteria, melanin, and hemocytes within 1 hour post-infection (Figures 4 and 5). These results were mirrored by quantification of *in vivo* melanization 3 hours post-infection (Figure 3) and *in vivo* melanization over several days post-infection (Figures 1 and 6(b)). Activation of the humoral response seemed to limit bacterial persistence and spread efficiently, as bacterial burdens of the hemolymph and other tissues were significantly decreased in larval samples infected with ΔMAM compared to samples from wild-type *Y. pseudotuberculosis*-infected larvae (Figure 2). Melanin production is emblematic of the insect humoral response. In the presence of a pathogen, hemocytes are triggered to release (de-granulate) their contents – which include inactive proPO localized within crystals alongside activators (chymo/trypsin) and substrates (mono- and di-phenols) – into the extracellular environment, resulting in the deposition of melanin on “non-self” entities [20]. The formation of melanized cellular aggregates is referred to as capsules or nodules (depending on the microbial target). As such, we observed partial protection when melanin was administrated to larvae prior to infection, providing further evidence that melanin is antimicrobial and/or limits bacterial spread by immobilizing bacteria in aggregates (Figure 7). However, our data also showed that activation, particularly hyperactivation, of humoral responses, caused significant collateral damage to the host. Infection induced extensive hemocytic death, which was further exacerbated by hyperactivation of melanization (Figure 2). This immune-mediated damage also manifested in larval morbidity (activity index) and mortality (survival), which were enhanced in larvae both that were RGD-treated and infected with ΔMAM, conditions that limit bacterial internalization by hemocytes (Figures 1 and 6). Additionally, administration of polymerized melanin, which does not require endogenous catalysis by the host, limited bacterial persistence without negatively impacting host viability (Figure 7), supporting the hypothesis that immune-mediated damage induced by melanin biogenesis, i.e. oxygenic radical production, contributed to pathogenesis.

The damage-response framework (DRF) first brought forward by Casadevall and Pirofski provides an integrated view of pathogenicity, whereby both bacterial traits and the host’s immune response affect virulence, reminiscent of the Jarisch-Herxheimer reaction [1,48]. *Yersinia* pathogenicity in vertebrates has been shown to be influenced by both bacteria-driven processes and host response-driven damage. In other words, depending on the extent of the host immune response, the overall outcome of infection can range from septicemia (if immune responses are too weak), to enterocolitis, to clearance, and even reactive arthritis (if immune responses are too strong) [1,2]. The DRF highlighted many other clinically important bacterial, viral, fungal, and protozoan infectious agents wherein pathogenicity cannot be captured as a function of bacterial virulence traits alone. This group include *Staphylococcus aureus, Influenza*, and *Coccidioides* [1].

For *Y. pseudotuberculosis*, mutations that decreased bacterial adhesion were associated with enhanced bacterial spread within the host, an exacerbated host immune response, and increased morbidity in rodent models [49]. It has been hypothesized that the partial loss of functional adhesins during *Yersinia* evolution may contribute to the increased ability of some strains to evade phagocytosis, thereby increasing pathogenicity [50]. However, no studies have systematically tested this hypothesis or investigated the link between *Yersinia* adhesion, phagocytic interactions, and immune-mediated damage. Our study suggests that the use of *G. mellonella* larvae as model host may allow us to address these open questions and to probe the full spectrum of microbe–host interactions. The *G. mellonella* model enables systematic testing that could help identify specific bacterial components and host innate immune traits that contribute to overall pathogenicity.

## Supplementary Material

Supplemental Material
