# Supplementary material for: Defective phagocyte association during infection of Galleria mellonella with Yersinia pseudotuberculosis is detrimental to both insect host and microbe
Source: Virulence. 2021 Feb 8;12(1):638–53. doi: 10.1080/21505594.2021.1878672 (PMC7889024; doi:10.1080/21505594.2021.1878672)
Supplement: Supplemental Material [file KVIR_A_1878672_SM8160.pdf]

Defective phagocyte association during infection of Galleria mellonella with Yersinia pseudotuberculosis is detrimental to both insect host and microbe by Anne Marie Krachler, Natalie Sirisaengtaksin, Pauline Monteith, C E Timothy Paine, Christopher J. Coates, Jenson Lim

Reference sequence (1): Y.ruckeri
Identities normalised by aligned length.
Colored by: identity

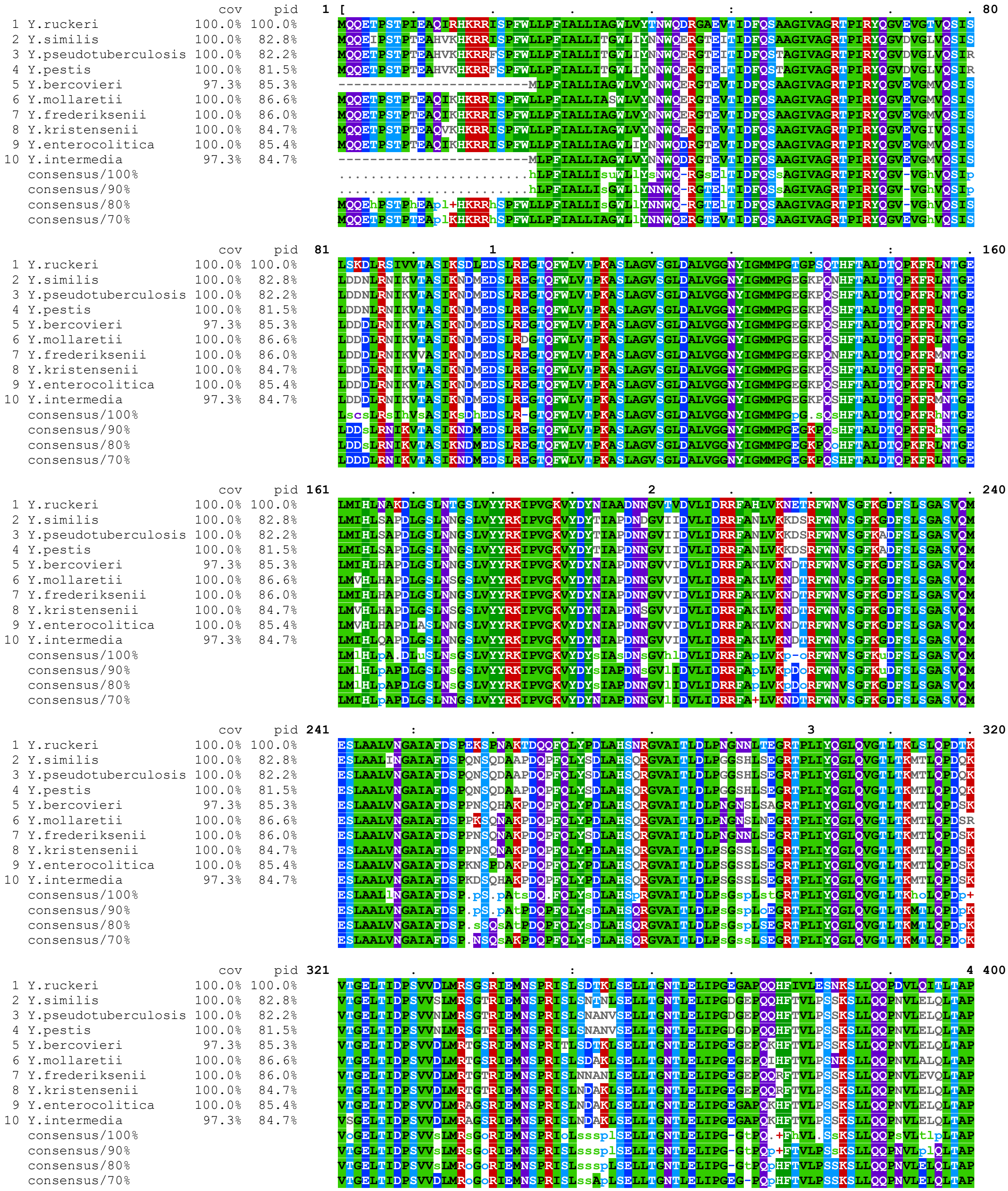

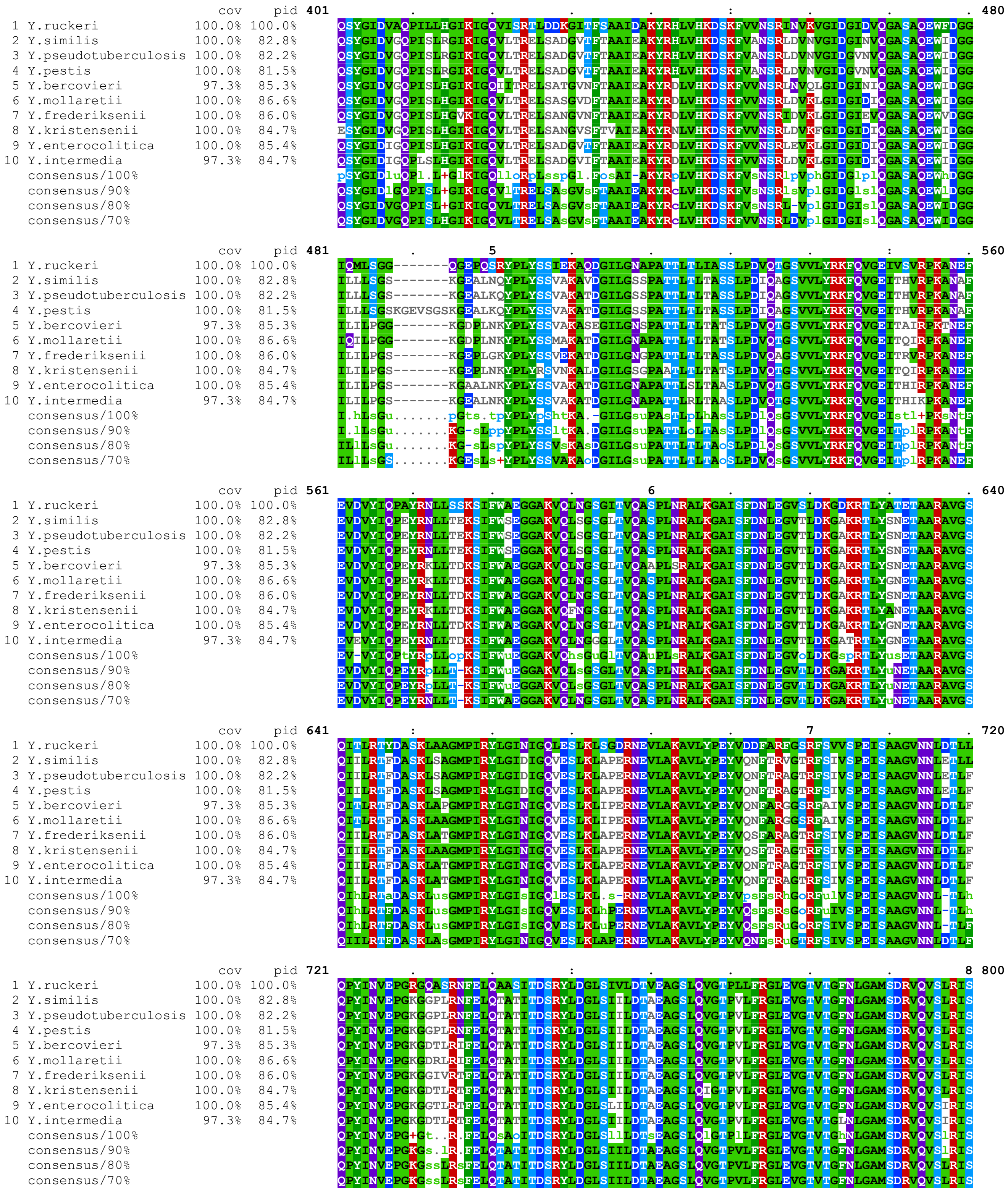

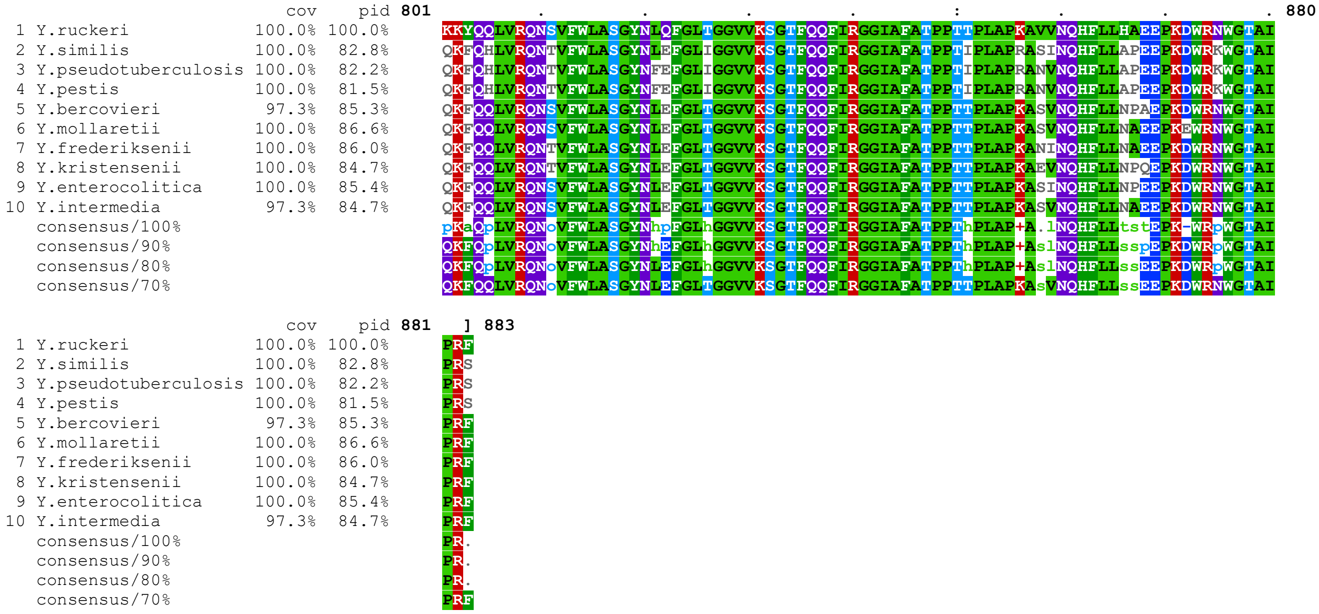


**Figure S1. Sequence alignments of MAM homologs from *Yersinia* spp.** Sequences from *Yersinia pseudotuberculosis* (ACA68070.1), *Yersinia pestis* (EFA49694.1), *Yersinia enterocolitica* (WP_083158656.1), *Yersinia kristensenii* (WP_145592019.1), *Yersinia bercovieri* (EEQ07651.1), *Yersinia frederiksenii* (WP_145508990.1), *Yersinia intermedia* (EEQ20374.1), *Yersinia mollaretii* (WP_145520873.1), *Yersinia ruckeri* (WP_145497088.1), and *Yersinia similis* (WP_145480659.1) were aligned using Clustal Omega (https://www.ebi.ac.uk/Tools/msa/clustalo/)[1] and the output file visualized using MView (https://www.ebi.ac.uk/Tools/msa/mview/).[2]

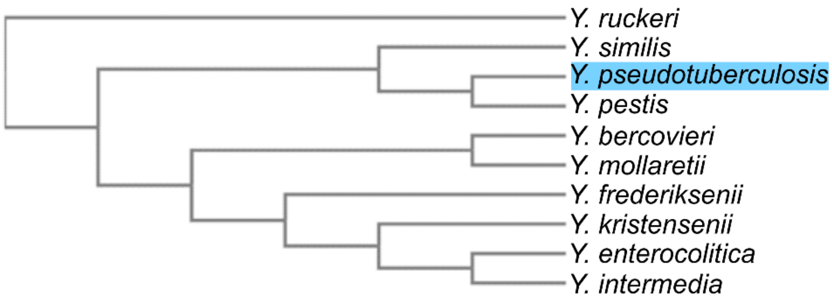


**Figure S2. Cladogram of MAM homologs from Yersinia spp.** based on multiple sequence alignment shown in Figure S1. *Y. pseudotuberculosis* MAM sequence (ACA68070.1) subject to this study is highlighted in blue.

TTCACTGAACCCCTGATCAGTATTCGCTTACTTGGCACACGTATTGATGCCAGTCTGTTAGCCGGGATTTGGCAAATGAGCCGT
CAAGGCGACCCCATCACAGCCAGCATGGTGGCATTTTGTGTCCTCGCGGCACCGGTAACCTTAACCTTCTCTATTCTATATCTG
CGTATTGGTAGCCGGATTGGCATAAATCTGCGCCCGATACTGCTTATATTGGAGCGACTGAAAGAGTGGGTCATGCTGGATATC
TATTTAATCGGCATGGCCGTCGCCTGCATTAAAGTCAAAGAATACGCTGACATCATGCCGGGTACAGGTCTGATAGCCTACCTG
GCCTTGACATTACTGAGTATTCTGACCTTGGTTCATCTGAATCTGGAGCAACTTTGGGAACGGTTTTATCCGCAAGAACAGCCTC
CAGGCCCGCAGGAAACACTGCGCGTTTGCTTATCGTGCCACTACACTGGACACCCCGATAGCCATGGCCGTTGTCCGCGTTGC
CATACACCTCTCCGACACCGCCGGCGTCATAGCCTACAGAAAACCTGGGCCGCATTGATTGCCGCTATCGTTTTGTTATTGCCC
GCCAACCTACTCCCAATATCAATCGTTTATGCCAACGGCGCACGGATAGAGGATACCATTTTCTCCGGTGTGGTTTCTTTGGCG
TCATCGGGTAATTTCCCTATTGCTGCCGTGGTCTTCATCGCCAGTGTATTGGTGCCGTTTACTAAAGTGATCGTGTTAATCACGT
TGCTGCTCAGTATTCACCTAAAGACACAACACAGCCTGAAAACCCGGATGCGTTTGCTGCGGTTGATCACCTGGATTGGTCGCT
GGTCAATGCTGGATCTCTTTGTTATCGCATTAATGATGTCGCTAATTAATCGCGATCAGCTTTTTTCCTTCACTATGGGGCCAGC
AGCCTTCTATTTTGGGTCTGCGGTTATTTTGACTATTCTTGCTGTTGAGTGGCTAGATAGCCGATTGATTTGGGATGCATATGCA
ACAGGAAACACCGAGTACACCGACTGAGGCTCATGTAAAACATAAACGCCGGTTCTCCCCCTTCTGGCTACTGCCTTTTATTGC
GCTACTGATCACGGGCTGGCTGATTTATAACAATTGGCAAGAACGTGGGACGGAAATCACTATTGATTTTCAGTCAACTGCTGG
AATTGTTGCTGGCCGCACACCCATCCGCTATCAAGGCGTGGATGTAGGTTTGGTTCAATCCATCAGATTGGATGATAACCTGCG
TAACATTAAAGTGACCGCCAGTATCAAAAATGATATGGAGGATTCACTACGTGAAGGTACCCAGTTCTGGCTAGTGACCCCCAA
AGCCTCTCTGGCCGGTGTTTCTGGATTGGATGCGCTGGTGGGCGGTAATTATATCGGCATGATGCCTGGTGAGGGTAAGCCGC
AAAGCCATTTCACTGCATTGGATACTCAGCCTAAATTCCGCCTGAATACAGGCGAATTAATGATTCATCTCTCTGCCCCTGACCT
CGGCTCACTCAATAACGGCTCGTTGGTTTACTATCGTAAAATTCCGGTTGGCAAAGTGTATGACTACACGATCGCCCCTGATAA
TAACGGGGTTATTATTGATGTCCTGATTGATAGACGATTTGCCAATTTGGTGAAAAAGGACTCTCGATTCTGGAATGTTTCTGGT
TTTAAAGCTGATTTCAGCCTAAGCGGTGCCTCGGTGCAAATGGAGAGTCTGGCGGCGCTGGTCAATGGCGCAATTGCTTTCGA
TTCCCCACAAAATAGCCAAGATGCCGCGCCAGATCAGCCTTTCCAACTTTATTCTGATTTAGCGCACAGTCAGCGCGGGGTGG
CCATCACCCTGGATTTACCAGGGGGAAGTCACTTGAGTGAGGGCCGTACCCCCCTGATTTATCAGGGGTTGCAAGTTGGTACT
TTGACTAAAATGACGCTTCAACCAGACCAAAAAGTGACGGGAGAGCTAACCATTGATCCCTCCGTAGTGAATTTGATGCGTAGT
GGCACACGCATTGAAATGAATAGCCCGCGGATCAGCCTCAGTAATGCCAACGTAAGTGAACTGCTTACTGGTAATACATTAGAG
CTGATCCCAGGCGACGGTGAGCCACAACAACATTTTACCGTACTGCCCAGCAGTAAAAGCCTGTTACAACAACCCAATGTCCTT
GAATTGCAACTCACCGCGCCACAAAGCTATGGCATTGATGTCGGCCAGCCTATTTCACTACGTGGTATCAAGATTGGTCAGGTG
TTAACCCGCGAACTGTCAGCCGATGGCGTCACCTTTACGGCCGCGATAGAAGCGAAATATCGCCATCTGGTGCACAAGGACAG
CAAATTTGTGGCAAACAGCCGCCTTGATGTCAACGTGGGGATTGATGGCGTTAACGTGCAAGGCGCCAGCGCACAAGAATGGA
TTGATGGCGGTATTCTGCTTTTATCAGGTAGCAAAGGCGAGGCACTGAAACAATACCCCCTTTACAGTAGCGTGGCAAAAGCAA
CTGATGGGATTTTAGGCAGTTCACCGGCAACGACCCTAACGCTAACGGCCAGCAGTTTGCCTGATATACAGGCCGGTTCTGTG
GTGTTGTACCGTAAGTTTCAGGTGGGTGAAATTACTCACGTACGCCCTAAAGCCAACGCCTTCGAAGTGGATGTTTACATTCAA
CCCGAGTATCGCAATCTACTAACCGAAAAGAGTATTTTTTGGTCAGAAGGGGGAGCAAAAGTACAACTGAGTGGGAGTGGCCTT
ACTGTACAAGCCTCACCGCTTAACCGGGCCTTAAAAGGGGCCATCAGCTTCGATAATCTAGAAGGCGTCACTCTGGATAAAGG
GGCCAAGCGGACATTGTATAGTAATGAAACGGCCGCACGGGCCGTGGGTAGCCAGATTATATTGCGTACATTTGATGCCAGTA
AACTGTCGGCGGGTATGCCAATCCGCTATTTGGGGATCGATATTGGCCAAGTTGAGTCGCTGAAGCTGGCACCCGAGCGTAAT
GAAGTCTTAGCCAAAGCCGTACTTTACCCAGAATATGTGCAAAACTTTACCCGCGCGGGTACCCGTTTCTCCATCGTCTCACCG
GAGATCTCGGCTGCAGGGGTCAATAATCTGGAGACCTTGTTCCAGCCCTACATTAATGTAGAGCCCGGTAAAGGGGGGCCCTT
GCGCAATTTTGAGTTGCAGACCGCCACCATTACTGACTCCCGTTATCTTGATGGCCTGAGCATCATACTGGATACCGCTGAAGC
GGGTTCGTTGCAAGTTGGGACGCCAGTCCTGTTCCGTGGGCTTGAAGTTGGCACTGTCACCGGTTTTAATCTGGGTGCAATGT
CCGATCGAGTGCAGGTATCACTGCGGATAAGCCAAAAGTTTCAGCATCTAGTAAGACAAAACACGGTATTTTGGCTAGCATCAG
GTTATAACTTTGAGTTCGGTCTGATAGGTGGTGTGGTGAAAAGCGGGACCTTCCAGCAGTTTATCCGTGGTGGTATCGCTTTCG
CTACGCCACCAACCATACCACTGGCACCGAGAGCCAATGTCAATCAGCACTTTTTGCTCGCCCCCGAAGAGCCTAAAGATTGG
CGAAAATGGGGAACAGCCATTCCACGTTCTTAAATAATTCATCAGGGTGGTTCGCCATAAGCTGCCCTGATGATTACCCGTGCT
CATGCCCTGCACAGCAATCGCCATTCTTATAAACCCTTTTTCTAAATTCCTGTAGCACTTACCGCTGTTTTCTTTTTTTATCTCTAA
ATTCGTCTCTTTATTTTCCATCATCTCTATTTTTCTTCGCCTATTTATTCAAAAGAGAAGCTAATCAATTTACTCACCCATCAATAAT
AAAGTCTTCATGTCGTTTTTTTGCTGAATGGATTCATATTAAATTTTTTTTTTTAAAAAAATAAAAATAATATTGAACCTAAAATAAA
TCAACATTAGGATAATGAAATTTATTATTTATTGCGTCTTGTTGGCGTTAATTAATCATTCCTTTCTCTAATGTTTAGCAACAAGAA
ATATTAGTTGCTTTACATTATTCTCTCAACAGCATCACGCAGGTTTCTTGGCGGTTAATTATAGGGTTAAGGGGGGGTAATGAGG
AAAACCTTATTTACTTTAAGTGTAATAGCCATATTACAGACAGCACCACACATAACCTATGCTGCTGCAACTACCGGTACACTGT
CATCAACACTGACCATTATCTCCGGCTGTTATATCAACGATGGGACCGGGTCTGGAAGCCTGAGTAATTTGGGAACCATAAACT
TCGGTAGTGTAGTGGCGCTATCCTCAGTAATTAACGTCGCATTCAGTGGCACACTGAGCGGGACTCTCAGTTTGTATTGTAGTG
CTAATACCGCGTATACCATCGCTATTGATAATGGGCTGTACAGCACGTCAGGACAGCGTCGGCTCCGGGGTGGGGCCGCAGC
ACCAACCACCTTTGAGTATGTTAATTATAATCTGTTCAAAGACAGTAGTTACAATCAGCCATGGAACGCAACCCCGACGACGGG
TGTTCAAAGTGGTACCGGCACCGGGATT

**Figure S3. Partial *Y. pseudotuberculosis* genome sequence. MAM (**ACA68070.1) coding sequence (yellow); -1kb fw and rev primer binding sites (blue); +1kb fw and rev primer binding sites (grey); MAM complementation primers (green); MAM promoter (sigma70) -10 and -35 elements (magenta) are highlighted.

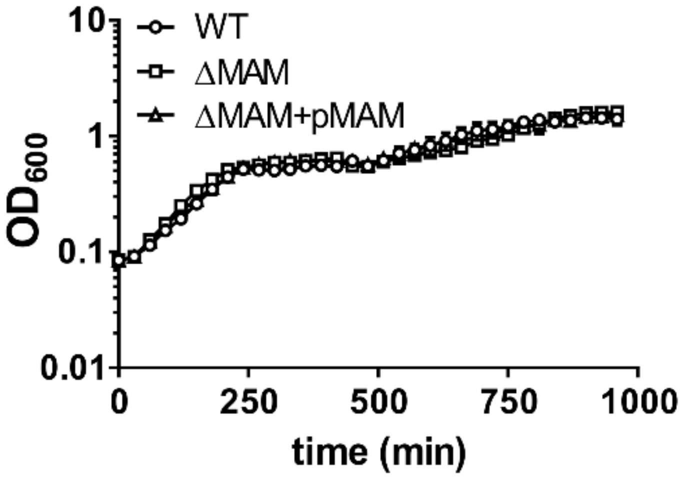


**Figure S4. Growth curves for *Y. pseudotuberculosis* strains used in this study.** *Y. pseudotuberculosis* YP126 wild type (○), ΔMAM (□), or ΔMAM containing a MAM complementation plasmid (Δ) were grown in LB at 37 °C, diluted into fresh LB to an $OD_{600}$ of 0.1 and $OD_{600}$ measured at 37 °C every 30 min for 16.5 h. Values are means ± s.e.m. (n=3). Statistical significance compared to YP126 WT was determined by ANOVA, and a Dunnett’s multiple comparisons test. No significant differences were found between the three strains.

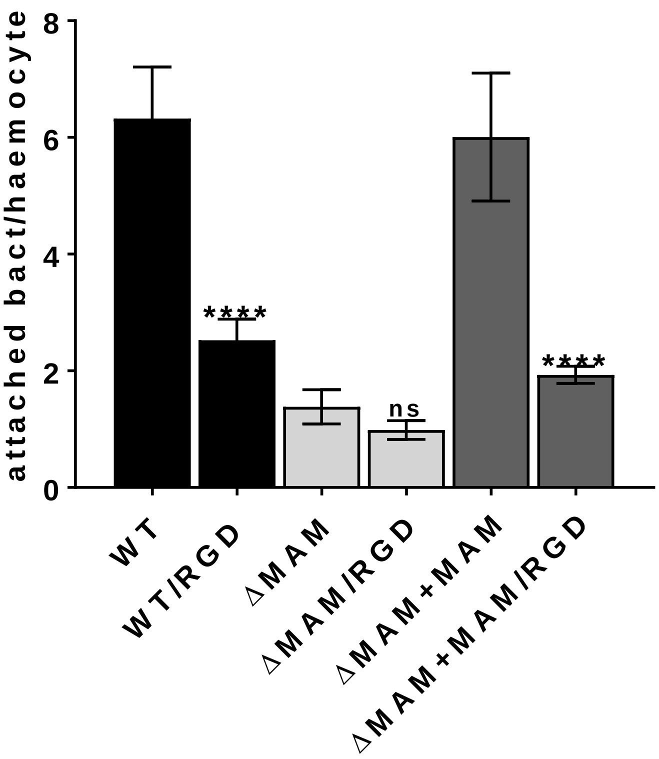


**Figure S5. Comparison of *Y. pseudotuberculosis* association with haemocytes with or without RGD peptide treatment.** Comparison and statistical analysis of datasets shown in Figures 5 and 7. Statistical significance between haemocyte association of each strain with or without RGD peptide treatment was determined by ANOVA, and a Dunnett's multiple comparisons test. (****) $p \leq 0.0001$, (ns, not significant) $p \geq 0.05$.

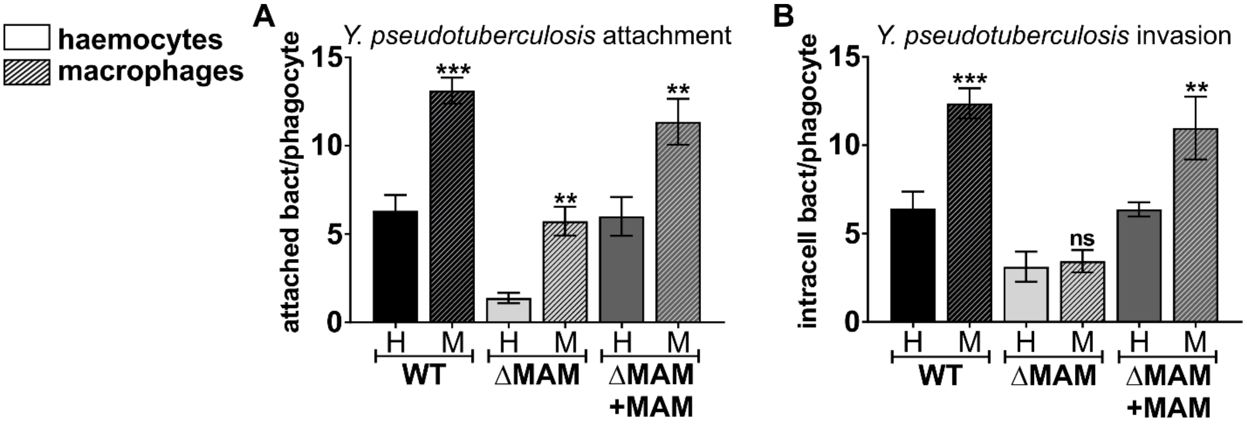


**Figure S6. Comparison of *Y. pseudotuberculosis* binding and invasion of *G. mellonella* haemocytes and mouse macrophages.** Side-by-side comparison of **(A)** bacterial association data from Figures 4E (haemocytes, filled bars) and 8M (macrophages, striped bars) and **(B)** bacterial invasion data from Figures 4L (haemocytes) and 8H (macrophages). Results are expressed as the mean ± s.e.m. of n=4 (haemocyte and macrophage association, macrophage invasion) or n=6 (haemocyte invasion) independent experiments. Significance between association of bacteria to haemocytes and macrophages, or invasion of haemocytes and macrophages was determined for each strain, using one-way ANOVA and a Dunnett's multiple comparisons test. (***) $p \leq 0.001$, (**) $p \leq 0.01$, (ns, not significant) $p \geq 0.05$.

## REFERENCES

1. Sievers F, Wilm A, Dineen D, Gibson TJ, Karplus K, Li W, et al. Fast, scalable generation of high-quality protein multiple sequence alignments using Clustal Omega. Mol Syst Biol 2011; 7:539.

2. Brown NP, Leroy C, Sander C. MView: a web-compatible database search or multiple alignment viewer. Bioinformatics 1998; 14:380-1.
